# Analysing the Relationship Between Immigrant Status and the Severity of Offending Behaviour in Terms of Individual and Contextual Factors

**DOI:** 10.3389/fpsyg.2022.915233

**Published:** 2022-06-15

**Authors:** Gloria Fernández-Pacheco Alises, Mercedes Torres-Jiménez, Paula Cristina Martins, Silvia María Vale Mendes

**Affiliations:** ^1^Department of Law and Criminology, Universidad Loyola Andalucía, Seville, Spain; ^2^Department of Quantitative Methods, Universidad Loyola Andalucía, Córdoba, Spain; ^3^Research Centre on Child Studies, Institute of Education, University of Minho, Braga, Portugal; ^4^Research Centre in Political Sciences, University of Minho, Braga, Portugal

**Keywords:** youth offending, migrant origin, self-reported data, integration policies, Portugal

## Abstract

**Background:**

Social inclusion is a context for both risk and protective factors of migrant youth delinquency. This study aims to shed light on the issue by comparing delinquency amongst native, first-generation, and second-generation immigrant youths in Portugal, a country located in the south of Europe, an area where research in this field is still scarce.

**Methods:**

The research is based on the International Self-Reported Delinquency (ISRD-3) dataset, which includes information on over 4,000 adolescents, who self-reported on their socio-demographic status, leisure activities, school and neighbourhood environment, family bonds, and self-control.

**Results:**

Nested Logistic Regression analyses showed that a young first-generation immigrant is twice as likely to commit a crime, with or without violence, as a young native born in Portugal. However, no differences were found regarding the prevalence of delinquency amongst second-generation immigrants and natives, which is likely due to the integration and cultural assimilation of the immigrant over time. Regarding the analysed risk factors, it was found that both structural and individual factors, identified by the theories of control, stress, as well as situational action theory, have a direct effect on the commission of juvenile crimes (both non-violent and violent). Moreover, this effect is significant in adolescents living in Portugal in general, both immigrants and natives. The most influential variable for both types of delinquent behaviour, with and without violence, is peer delinquency, followed by low morality and self-control.

**Conclusion:**

These findings have relevant policy implications and are useful for evidence-based interventions aimed at promoting migrant adolescent well-being and targeting host countries’ performance.

## Introduction

Social inclusion is an issue of international scope with national implications whose political management is fundamental amongst the challenges presented by the UNESCO Sustainable Development Goals (Carens, 2004). This has been recognised by the United Nations amongst its priorities for the 2030 global agenda. Specifically, Goals 10 and 16 directly address social inclusion in the ambition to reduce inequality within and amongst countries. Failure to ensure inclusive societies is identified as a causal factor of violence and insecurity that curtails sustainable development (UNESCO, 2022). The counterpoint to the sustainability perspective is to be found in some political movements that have emerged in developed countries in recent decades, which emphasise the relationship between immigration and offending behaviour as one of their main arguments. Contributing to this debate by providing scientific evidence to analyse whether such a relationship between offending behaviour and immigration really exists and, if it does, to identify the main factors that lead to it, is precisely the main objective of this research.

This is not a new issue, as differences in involvement in offending behaviour between migrants and the native population have been a major topic of discussion in criminology since European countries began to receive large numbers of foreigners, a trend that was driven by job seeking in the 1950 and 1960s. This phenomenon has been accompanied by a tightening of migration policies in Europe (Tonry, 1998; Killias et al., 2004). Nevertheless, self-reported delinquency amongst adolescents in Europe does not clearly show a higher rate of immigrants involved in antisocial behaviour and delinquency than natives (Junger-Tas, 2001). On the one hand, the existence of a significant correlation between immigrant origin and crime has long been recognised in the scientific literature (Killias, 1989; Junger-Tas, 1997, 2001; Marshall, 1997; Kardell and Martens, 2013; Salmi et al., 2015; Bovenkerk and Fokkema, 2016). Other studies, however, have found no significant differences in the propensity for offending behaviour of native youths and that of immigrant youths (Junger-Tas, 1997; Torgersen, 2001). Moreover, the dynamics of this relationship (between immigrant status and offending behaviour) remains unclear (Salmi et al., 2015; Serrano-Maillo, 2018).

These contradictory results suggest that further research is needed to fully understand if there are differences between the prevalence of delinquency in immigrant youths and that in native youths, and the mechanisms that might contribute to such differences. Similarly, it would be worthwhile to examine whether those factors that are recognised as predictors of offending behaviour in central and northern Europe are applicable in southern Europe. Regarding these predictors, whilst many studies have only focused on contextual or individual factors, the combination of both types should be considered in the prediction of offending behaviour (Duran-Bonavila et al., 2017). To that end, the present research attempts to understand the influence of both contextual—or structural—factors (such as family structure, neighbourhood and school disorganisation, and peer delinquency) and individual factors (such as morality and self-control) on delinquent youths offending in Portugal, differentiating between their origin (native or immigrant). In turn, an analysis of immigrant status has been carried out distinguishing between first- and second-generation youths. In addition, a separate analysis has been conducted of violent and non-violent offending behaviour in both native and immigrant youths in order to ascertain if different patterns pertain to different types of crime.

Finally, youth age and gender have also been included as predictor variables of offending behaviour as the influence of both variables on the propensity to juvenile delinquency has been empirically demonstrated in criminological research (Grasmick et al., 1993; Sampson and Laub, 1997; Moffitt and Caspi, 2001; Torgersen, 2001; Ribeaud and Eisner, 2010; Bovenkerk and Fokkema, 2016).

## Portugal as an Immigrant Country

Portugal has changed radically over the past decades, from a country from which many people emigrated in order to find work in other European countries, to a country of choice for immigration (Casqueira, 2006). Although the total number of immigrants remains relatively small, the new influx of people implied the end of a culturally homogeneous society. Immigration to Portugal was, until the mid-1990s, an inheritance from the colonial period. Most immigrants came from Cape Verde, Angola, or Brazil. Since the late 1990s, a boom in construction has ushered in a new flow of immigrants from Eastern Europe (mostly Ukrainians), Brazil, and the former Portuguese colonies in Africa (Casqueira, 2006). Their communities currently constitute the largest immigrant groups in Portugal. Official data from the Portuguese Foreign Office (Portdata, 2019) show that the foreign population living in Portugal grew steadily from the end of the 20th century until 2009, when it began to fall. Then in 2015 it began to rise again until it reached its historical peak in 2018 with 477,472 foreign residents (4.7% of the total population). The immigrant population of Portugal originates from Brazil (25.5%), Cape Verde (12.1%), Ukraine (11.0%), Romania (8.7%), Angola (4.9%), Guinea-Bissau (4.2%), and the United Kingdom (4%). It is worth noting that the immigrant population is not evenly distributed throughout the country, with concentrations of ethnic minorities mostly found in urban areas (Casqueira, 2006).

## Explaining the Juvenile Delinquency–Immigrant Nexus in Europe

The relationship between immigration and juvenile delinquency is one of the most recurrent topics in criminology. Theoretical tradition in this discipline has emphasised sociological and structural factors, such as family disorganisation, lack of rules, and social marginalisation, as precursors to criminality. According to the social disorganisation theory (Shaw and McKay, 1942), neighbourhoods with poor living standards, poverty and population instability suffer from high crime rates. Unemployment and economic deprivation force immigrants to settle in disadvantaged residential areas, where residential turnover weakens social bonds and social control. The additional disadvantage of poorer schooling in these areas has also been researched as a risk factor for delinquency (Eklund and Fritzell, 2014; Pauwels and Svensson, 2015), as has the high concentration of immigrant youths in urban neighbourhoods (Kardell and Martens, 2013). According to the theory of control (Hirschi, 1969), immigrants’ lack of connection with their host society, as well as the break with their culture of origin, favours the development of criminal behaviour. Moreover, a lack of parental supervision and weak family bonds increase the likelihood of delinquent behaviour (Gottfredson and Gottfredson, 2013).

One of the factors that have repeatedly been found to contribute to the development of offending behaviour amongst adolescents is peer influence, because peers serve as role models for behaviour (Moffit, 2006; Titzmann et al., 2008). Peer-oriented leisure activities are related to a higher risk of delinquency, because lack of structure and little supervision by adults in the contexts where such activities take place provide opportunities for delinquent behaviour (Mahoney et al., 2004). Several authors have identified lifestyle risk and peer delinquency as predictors of offending behaviour (Wikström and Butterworth, 2006; Wikström and Svensson, 2008; Marshall and Enzmann, 2012; Schils and Pauwels, 2016; Pauwels, 2018).

In addition, individual factors configure a person’s crime propensity, according to the situational action theory (Wikström and Butterworth, 2006), which is one of the most tested contemporary theories. Morality plays an important role in crime propensity. Individuals may consider an act to be good according to their own cultural and moral rules, even though the legal system may forbid it and have declared it illegal. Thus, morality has been considered a relevant individual risk factor for juvenile delinquency (Svensson et al., 2010; Wikström et al., 2012).

Low self-control is another relevant individual risk factor that explains active offending (Svensson et al., 2010; Bruinsma et al., 2015; Jansen et al., 2016). According to theory of control of Gottfredson and Hirschi (1990), self-control is a multi-faceted trait that focuses on the ability to defer the immediate gratification of desires when such gratification results in long-term negative consequences.

The accumulation of all the above mentioned risk factors (neighbourhood and school disorganisation, lack of parental control, peer delinquency, and low levels of morality and self-control) increases the likelihood of developing offending behaviour, as is stated by life-course theory of crime of Sampson and Laub (1997). These risk factors should be considered “turning points” in the development of offending trajectories (Pratt, 2016).

## Prior Research on Juvenile Delinquency Using Self-Reported Data

Studies conducted to analyse the differences in the prevalence of offending behaviour between native and immigrant youths have generated contradictory results. For instance, some studies based on self-reported delinquency in Europe found that immigrant youths had either similar or lower crime prevalence rates compared to the native population (Junger-Tas, 1997; Torgersen, 2001). Other studies conducted in northern European countries, on the other hand, revealed higher rates of violent delinquency in second-generation immigrants in Germany (Enzmann et al., 2010), Switzerland (Killias et al., 2010; Ribeaud and Eisner, 2010) and Sweden (Svensson and Shannon, 2020). In Norway, a study found some differences in patterns of self-reported delinquency amongst adolescents with respect to immigrant status, gender and country of origin (Torgersen, 2001). Titzmann et al. (2008) confirmed that in Germany, the strength of association for variables such as parental knowledge, peer delinquency, and family violence differed between first-generation immigrant and native German adolescents, although the predictors of delinquency did not differ between second-generation immigrant and native adolescents. Therefore, as there is no clear evidence for the overall differences in crime rate between immigrant and native youths, further research is needed. Furthermore, only a few studies of this issue using self-reported delinquency have been conducted in southern European or Mediterranean countries (Sobral et al., 2010; Gatti et al., 2013). In Portugal, juvenile delinquency has been analysed in depth from a psychological and intervention perspective—with institutionalised male juveniles (Pinto et al., 2015) and young people overall (Cardoso et al., 2015)—but not from the perspective of immigrant youth involvement in offending.

In the present study, we attempt to analyse in detail the differences in delinquency involvement between native and immigrant youths residing in urban areas of Portugal. To achieve this general objective, and to summarise all the information analysed so far, this research pursues the following objectives (i) to analyse whether there are differences in the probability of developing delinquent behaviour between native and immigrant youths, distinguishing, in the latter group, between first and second generation; (ii) to study whether the individual and contextual risk factors identified by various theories as predictors of the development of delinquent behaviour in young people from northern and central European countries are also applicable in southern countries such as Portugal; and (iii) to determine whether there are differences between the influence exerted by these risk factors according to the seriousness of the crime, distinguishing between violent and non-violent offences.

## Methodology

### Sampling and Data Collection

For generalisation purposes, a stratified sampling was conducted in two waves: by city and by school grade. First, schools in three Portuguese cities—Lisbon (25%), Porto (27%), and Braga (48%)—were randomly selected, and then 7–12th grade classes were likewise randomly selected. Survey administration took place during a class session supervised by at least one research assistant. Oral consent from students was obtained after written and oral information on the study’s objectives was provided. Anonymity and confidentiality were ensured. Research assistants answered participants’ questions to ensure that study’s objectives, consent, and questionnaire were well understood.

Having received previous approval from the Portuguese Data Protection Agency, the Ministry of Education, and from the Ethical Committee of University of Minho, the questionnaire was administered, whenever possible online. Paper administration was used when no computers were available to administer the survey or when problems with web connections arose. Data collection was carried out from October 2015 to June 2016.

Participants included 4,124 students in grades 7–12, aged 12–18 years. These were recruited from 80 schools in three small-to-large-size cities in Portugal: Braga, Porto, and Lisbon. Of these, some questionnaires were excluded due to incomplete or invalid information. In total, 4,009 questionnaires were initially valid for the analysis (the descriptive statistics are presented in Table 1).

**Table 1:** Sample composition (*N* = 4,009).

Characteristics	*N*	Proportion (%)
*Gender*		
Male	1,865	46.5
Female	2,144	53.5
*Age*		
12–15 years old	2,172	54.2
16–18 years old	1,837	45.8
*Birthplace*		
Portugal	3,818	95.2
Brazil	50	1.2
Ukraine	14	0.3
Spain	6	0.1
Others	121	3.2
*Immigrant status*		
Native	3,169	79
1st G	191	4.8
2nd G	649	16.2
*Offending behaviour*		
No	2,626	65.5
Yes	1,383	34.5

Table 1 shows that both males and females were represented in the sample (with a slightly greater proportion of females—53.5%). Age is ranged from 12 to 18 years. The majority was born in Portugal (95.2%), including both natives (79%) and second-generation immigrants (16.2%), that is, adolescents who were born in Portugal but at least one of their parents is a foreigner. The remaining youths were 191 first-generation immigrants (adolescents who were born in a country other than Portugal—1st G), Brazil being the most popular country of origin.

As we can observe in Table 1, in most cases (63%) within the small group of first-generation immigrants (191) the country of origin was not specified. This fact prevents the analysis of the influence of culture of origin on the probability of committing criminal behaviour, having to consider the immigrant group as a whole.

Regarding offending behaviour, more than half of the students claimed not to have developed any such type of behaviour (65.5%).

### Instrument

The data are individual-level data based on the International Self-Reported Delinquency Questionnaire (ISRD-3) that was validated and administered by the authors of this study. The ISRD-3 is a standardised self-report questionnaire on juvenile delinquency and victimisation. The questionnaire includes items of a sociodemographic nature, as well as on family, school, victimisation, leisure and peers, neighbourhood, attitudes, and values (pro-social, self-control, and neighbourhood), offending, substance use, the strength of norm transmission, procedural justice, and gang membership.

Structural factors are measured in the ISRD-3 through questions on family structure, neighbourhood environment, school environment, and the students’ leisure time or unsupervised activities, specifically on their frequency of going out, the kinds of activities they engage in, and their time spent hanging out with friends, in line with Steketee (2012). Morality and the strength of norm transmission from family, school, and peers is measured in the ISRD-3 using two types of vignettes: one measures the “subjective norms” of the respondents by asking how they view the norms of relevant others—parents, teachers, and peers—in terms of unethical (but legal) behaviour motivated by personal gain (enrichment); the other measures the same with regard to illegal behaviour (theft). Combining these two measurements creates a scale for the strength of norm transmission. A shorter version of the Grasmick et al. (1993) scale has been added, this shorter version including the following constitutive elements of low self-control: impulsivity, quick-temperedness, self-centeredness, a preference for simple tasks, and physical risk-seeking activity.

### Measures of Risk Factors

The overall variable to predict was the probability of developing an offending behaviour, distinguishing whether this was of a violent or non-violent nature. Then, the variables used as predictors (considered “the risk factors”) were divided into two groups: *structural* (family structure, parental social control, peer delinquency, neighbourhood disorganisation, and school disorganisation) and *individual* factors (morality and self-control). Immigrant status was included in the analysis to distinguish between first-generation, second-generation, and native.

The survey items used to operationalise selected risk factors were coded on a five-point Likert scale in ascending order (the higher the score for the factor, the worse the situation of the participant). The operationalisation of each item in each variable was performed using factor analysis, specifically, principal component analysis. The results proved the adequacy of the factor analysis (Barlett’s test of sphericity significance was 0.000 and the Kaiser-Meyer-Olkin sample adequacy measure was greater than 0.75). For each construct (or “risk factor”), two components were always obtained that explained at least 60% of the variance. The scores of each participant for each factor were obtained using the regression method. Thus, the linear combination of these components’ scores, weighted by their contribution to the variance, became the final value of each construct or “risk factor.” The interpretation of the meaning of each extracted component, as well as its contribution to the total variance, is shown in Table 2.

**Table 2:** Reliability analysis and the components of each risk factor.

Factor (Cronbach’s alpha and number of items)	Component 1 (Meaning a % of variance contribution)	Component 2 (Meaning a % of variance contribution)
Parental control α = 0.73 Items: 5	Parents are interested in knowing what their children do when they go out (48.753%)	Parents check that the children fulfil their obligations (20.431%)
Neighbourhood disorganisation *α* = 0.78 Items: 9	Disorganised environment in the neighbourhood (34%)	Social control by neighbours (29%)
School disorganisation *α* = 0.72 Items: 8	Attachment to school (35%)	Disorganised environment at school (26%)
Peer delinquency *α* = 0.66 Items: 5	Peers’ violent offending behaviour (46%)	Peers’ non-violent offending behaviour (21%)
Morality *α* = 0.72 Items: 7	Opinion about serious criminal behaviour (39%)	Opinion about less serious criminal behaviour (55%)
Self-control *α* = 0.83 Items: 9	Risk taking (43%)	Impulsivity (15%)

In turn, Figure 1, in the Appendix, includes information on the description, scale and, if applicable, recoding of the ISRD-3 questionnaire variables considered in the analysis.

### Immigrant status

Immigrant status was explained through two binary variables—first- and second-generation—using native youth as the reference category.

### Structural Factors

Structural influence was measured using the following factors: family structure, neighbourhood disorganisation, school disorganisation, and peer delinquency (Appendix).

*Family structure* was dichotomised into the nuclear family (that is, both biological parents are present) and other family models. The *family’s financial circumstances* were based on the respondents’ evaluation of their family’s situation compared with that of other families; it was dichotomised into “average or better” than average financial circumstances and “below average” financial circumstances. We also asked if the respondent’s mother and father were employed.

The level of *neighbourhood disorganisation* was measured by the question, “How much do you agree or disagree with the following statements about your neighbourhood?” The question comprised nine items (*α* = 0.78) related to the criminal environment of the neighbourhood (such as the sale of drugs, fights, and graffiti) and the integration and social interaction of the neighbours (such as if they know, trust and get along well with each other—see Table 2).

The level of *school disorganisation* was measured by a set of eight items (*α* = 0.72) related to the respondent’s level of agreement with statements about their attachment to school (for example, if they like to go to school, if they find school interesting) and the criminal environment at their school (the sale of drugs, fights, and so on).

The *peer delinquency* factor was formulated in the survey by the question, “Young people sometimes engage in illegal activities. Do your friends usually engage in any of the following activities?,” followed by five items (*α* = 0.66) reflecting the following events: the consumption of soft or hard drugs, shoplifting, burglary, mugging, and assault.

### Parental Control

Parental control was measured using a five-item scale (*α* = 0.73), with questions such as whether the parents know where and with whom their children spend their leisure time and if they check that their children fulfil their obligations (Appendix).

### Individual Factors

From an individual perspective, *moral* values and *self-control* were included for analysis.

*Morality* was measured by a question (comprising seven items, *α* = 0.72) that sought to analyse students’ perceptions regarding the severity of several different criminal activities. The perceived severity of the analysed behaviours was measured through the evaluation of actions such as “Lie, disobey or talk back to adults such as parents and teachers,” “Steal something small such as a chocolate bar from a shop,” and “Hit someone with the intention to hurt that person” and “Break into a building to steal something.”

As is mentioned above, Grasmick et al. (1993) listed the constitutive elements of low *self-control*, a shortened version of the scale being used in the ISRD-3 (Q6.5). In total, nine items were included (*α* = 0.83).

### Offending Behaviour

The dependent variable includes information on the participant’s offending behaviour and is based on 13 offence items: graffiti writing/painting, destruction of property, shoplifting, stealing at school, stealing a bicycle, stealing a motor vehicle, stealing something off or from a car, burglary, bullying, taking part in a fight, beating somebody up, carrying a weapon, and selling drugs. The participants were specifically asked if they had committed any of the aforementioned offending behaviours in the previous year and were then clustered into three groups: those who had not committed any offence (2,626, 65.5% of the sample); those who had committed non-violent offences, such as vandalism, shoplifting and burglary (684, 17.1% of the sample); and those who had committed violent offences in the last year, such as robbery, extortion, group fighting, assault, and animal cruelty (699, 17.4% of the sample). Therefore, it can be stated that with respect to respondents who claimed to have engaged in criminal behaviour, they were equally distributed between violent and non-violent offences.

### Age and Gender

Since it is known that *age* and *gender* influence the development of offending behaviours (Sampson and Laub, 1997; Torgersen, 2001; Ribeaud and Eisner, 2010; Bovenkerk and Fokkema, 2016), they were included in the analysis as regressors. Some authors claim that average gender differences in antisociality (i.e., higher offending rates for males than for females) often begin in mid-adolescence and persist into early adulthood (Moffitt et al., 2001). Gender was coded with the value 0 for female and 1 for male. As for *age*, since there is a wide age range in the sample (12–18 years old), in which offending behaviour typically varies substantially, the sample was divided into two groups using the median age (15 years old), which coincides with the mid-adolescent stage when there is a rapid increase in the crime rate (Blonigen, 2010). Age was coded with the value 0 being taken for younger than or equal to 15, and 1 for the rest.

### Analysis

In order to examine the association of selected risk factors in offending behaviour, two nested logistic regression models (LRs) were run (one for each type of offending behaviour—non-violent and violent). In both LR models, the reference category would be represented by those participants who did not develop any offending behaviour.

Given the difficulty of interpreting the influence of quantitative predictor variables such as all those included in Table 2 (Neighbourhood and school disorganisation, parental control, etc.) on the response variable of a Logistic Regression (LR), they were categorised into three levels according to the terciles of their distributions, each with a different level of risk associated: high, medium, and low, the latter being the reference category (see Figure 1 of the Appendix).

To measure how the initial effect of migrant status (first and second generation) on the development of offending behaviour is attenuated when risk factor variables are incorporated, the Stata command *khb,* developed by Karlson, Holm and Breen, was used (Breen et al., 2013). This method allows an unbiased decomposition of the total effect in a non-linear context since, as is known, the decomposition of the total effect into direct and indirect mediated parts, using nested non-linear models, is problematic due to the problem of scale identification (Mood, 2010). In the logistic regression models, the importance of the change in the immigrant status coefficient between successive models was evaluated using the abovementioned method (khb).

Previous to the LR analysis, a preliminary study was carried out to ratify the influence of the control variables on the likelihood of developing an offending behaviour (non-violent or violent). Specifically, several hypotheses were tested in which the null hypothesis assumed the equality of participant proportions that had developed an offending behaviour in each immigrant status, age range, and gender. The confidence intervals estimated for the difference between these proportions are shown in Table 3.

**Table 3:** CI for the differences between the proportion of participants in offending behaviour according to immigrant status and offence severity.

Offending behaviour	Answer	Natives	1st G	2nd G	Total
Non-violent	No	79.7%	71.2%	79.6%	2,626
	Yes	20.3%	28.8%	20.4%	684
	Total	2,614 (100%)	146 (100%)	550 (100%)	3,310
Violent	No	79%	69.8%	81.6%	2,626
	Yes	21%	30.2%	18.4%	699
	Total	2.639 (100%)	149 (100%)	537 (100%)	3.325
CI (p_1_–p_2_)^*^ (“Yes” answer)		Natives—1st G	Natives—2nd G	1st G—2nd G	
Non-violent		CI (−0.16; −0.01)	CI (−0.038; 0.036)	CI (0.003; 0.165)	
Violent		CI (−0.17; −0.02)	CI (−0.010; 0.060)	CI (0.037; 0.190)	

* Highlighted significant differences (*p* < 0.05).

## Results

According to the results of the Confidence Intervals (CI) estimated for the difference between proportions (see Table 3), the proportion of participants who said they had engaged in some form of offending behaviour (violent or non-violent) was significantly higher for 1st G than for the other two groups (natives and 2nd G)^1^ but no significant difference was found between that proportion for natives and for 2nd G.

Regarding age, as expected, a greater proportion of participants who had engaged in some form of offending behaviour (violent or non-violent) was found in the oldest group, that is, those older than 15 (confidence intervals—CIs—are shown in Table 4). Consequently, it was deemed necessary to include age in the analysis.

**Table 4:** CI for the differences between the proportion of participants in offending behaviour according to age (younger-older) and offence severity.

Offending behaviour	Answer	Age ≤ 15 (p_1_)	Age > 15 (p_2_)	Total	Confidence interval (p_1_–p_2_)^*^ (“Yes” answer)
Non-violent	No	86%	71%	2,626	
	Yes	14%	29%	684	CI (−0.182; −0.125)
	Total	1,863	1,447	3,310	
Violent	No	84%	72%	2,626	
	Yes	16%	28%	699	CI (−0.145; −0.088)
	Total	1,910	1,415	3,325	

* Highlighted significant differences (*p* < 0.05).

In relation to the respondents’ gender, the CI showed a higher rate of offending behaviour in males (Table 5), particularly in violent offences.

**Table 5:** CI for the differences between the proportion of participants in offending behaviour by gender (male–female) and offence severity.

Offending behaviour	Answer	Male (p_1_)	Female (p_2_)	Total	Confidence interval (p_1_–p_2_)^*^ (“Yes” answer)
Non-violent	No	76%	82%	2,626	
	Yes	24%	18%	684	CI (0.033; 0.089)
	Total	1,406	1,904	3,310	
Violent	No	70%	87%	2,626	
	Yes	30%	13%	699	CI (0.140; 0.196)
	Total	1,525	1,800	3,325	

* Highlighted significant differences (*p* < 0.05).

Our results justify the consideration of *immigrant status*, as well as *age* and *gender*, as predictors of offending behaviour.

Tables 6, 7 display the results from the nested logistic regression models by using offending behaviour (non-violent and violent, respectively) as the response. In Models 1–4, factors that could explain the difference between the rates of offending behaviour for immigrants—distinguishing between 1st and 2nd generation—and natives were included progressively. The results of the models are reported as odds ratios (ORs).

**Table 6:** Logistic regression models predicting non-violent offending behaviour.

Predictor	Model 1	Model 2	Model 3	Model 4
*Immigrant status*				
Native	1	1	1	1
1st Generation	1.68^*^	1.78^*^	1.80^*^	2.01^**^
2nd Generation	1.01	1.03	1.00	0.99
*Age*				
<=15	1	1	1	1
>15	2.785^***^	1.296^*^	1.284^*^	1.411^**^
*Gender*				
Female	1	1	1	1
Male	1.611^***^	1.794^***^	1.838^***^	1.778^***^
*Family structure*				
Nuclear family		1.00	1.00	1.00
Other situation		1.04	1.01	1.06
*Family’s financial circumstances*				
Average or better		1.00	1.00	1.00
Below average		0.98	0.92	0.88
*Parents’ employment*				
Father working		1.00	1.00	1.00
Other situation		1.52^*^	1.49^*^	1.46
Mother working		1.00	1.00	1.00
Other situation		0.98	0.94	0.96
*Parents’ social control*				
High		1.00	1.00	1.00
Average		1.41^*^	1.35	1.26
Low		1.98^***^	1.88^***^	1.61^**^
*Peer delinquency*				
Low		1.00	1.00	1.00
Average		3.75^***^	3.68^***^	3.29^***^
High		13.24^***^	12.12^***^	11.05^***^
*Neighbourhood disorganisation*				
Low			1.00	1.00
Average			1.15	1.05
High			1.56^**^	1.36^*^
*School disorganisation*				
Low			1.00	1.00
Average			0.81	0.75
High			1.20	1.01
*Morality*				
Strong				1.00
Average				1.70^**^
Weak				2.12^***^
*Self-control*				
High				1.00
Average				1.12
Low				2.02^***^
*Pseudo R^2^*	*0.0515*	*0.2196*	*0.2274*	*0.2533*
*Khb 1st Generation (sig. Indirect effect)*		*0.216*	*0.244*	*0.393*
*Khb 2nd Generation (sig. Indirect effect)*		*0.566*	*0.684*	*0.660*

* *p* < 0.10;

** *p* < 0.05;

*** *p* < 0.001.

**Table 7:** Logistic regression models predicting violent offending behaviour.

Predictor	Model 1	Model 2	Model 3	Model 4
*Immigrant status*				
Native	1.00	1.00	1.00	1.00
1st Generation	1.87^**^	1.83^**^	1.86^**^	2.13^**^
2nd Generation	0.88	0.91	0.90	0.86
*Age*				
<=15	1	1	1	1
>15	2.262^***^	1.094	1.116	1.176
*Gender*				
Female	1	1	1	1
Male	2.521^***^	2.754^***^	2.697^***^	2.512^***^
*Family structure*				
Nuclear family		1.00	1.00	1.00
Other situation		1.30^*^	1.25^*^	1.30^*^
*Family’s financial circumstances*				
Average or better		1.00	1.00	1.00
Below average		1.07	0.99	0.97
*Parents’ employment*				
Father working		1.00	1.00	1.00
Other situation		2.72^***^	2.56^***^	2.62^***^
Mother working		1.00	1.00	1.00
Other situation		1.05	0.97	0.95
*Parents’ social control*				
High		1.00	1.00	1.00
Average		1.23	1.19	1.13
Low		1.85^***^	1.70^**^	1.50^**^
Peer delinquency				
Low		1.00	1.00	1.00
Average		4.25^***^	3.86^***^	3.43^***^
High		11.62^***^	9.36^***^	8.16^***^
*Neighbourhood disorganisation*				
Low			1.00	1.00
Average			1.40^*^	1.29
High			1.74^**^	1.35^*^
*School disorganisation*				
Low			1.00	1.00
Average			1.65^**^	1.52^**^
High			2.31^***^	1.86^**^
*Morality*				
Strong				1.00
Average				1.37^*^
Weak				1.93^***^
*Self-control*				
High				1.00
Average				1.06
Low				2.17^***^
*Pseudo R^2^*	*0.0631*	*0.2289*	*0.2489*	*0.2751*
*Khb 1st Generation (sig. Indirect effect)*		*0.083*	*0.090*	*0.211*
*Khb 2nd Generation (sig. Indirect effect)*		*0.450*	*0.569*	*0.617*

^*^p < 0.10;

** *p* < 0.05;

*** *p* < 0.001.

Regarding non-violent offending behaviour (Table 6, Model 1), where only gender and age are included, 1st generation immigrant youths are 1.7 times more likely to commit a non-violent offence. However, 2nd generation immigrant youths showed no greater risk of engaging in non-violent offending behaviour than natives (the OR was non-significant). As expected, those over 15 years of age and males were more likely to develop delinquent behaviour. The only family structure factor that showed a significant (but weak) influence on the likelihood of developing a non-violent offending behaviour was *father not working* (Model 2 vs. Model 3). However, the effect of this factor became non-significant when individual factors (morality and self-control) were included (Model 4). It appears that socioeconomic factors cannot explain a greater participation in non-violent offending behaviours. However, *low parental control* does have a significant influence on the development of non-violent offending behaviours, although this influence is reduced as other variables are incorporated into the model (1.98 in Model 2 vs. 1.61 in Model 4). *Peer delinquency* (at an average and a high level) is the factor with the greatest influence on the development of non-violent offending behaviours (the OR was over 11 in the case of high-level peer delinquency in Model 4). Neighbourhood disorganisation was influential but not school disorganisation. Nevertheless, low morality and self-control contributed significantly to the explanation of non-violent offending behaviours.

In the final model (Model 4), which includes all the explanatory factors, 1st generation immigrant youths are twice as likely to commit a non-violent offence (the OR was 2.01). The inclusion of the analysed explanatory risk factors, especially *peer delinquency*, improved the model’s goodness of fit (the pseudo R^2^ increased from 0.05 to 0.25). Therefore, these factors are directly associated with the risk of committing a non-violent offence.

However, the OR for the 1st generation immigrant variable did not decrease when additional risk factors were added to the model (and even increased a little). The influence of being a 1st generation immigrant on the development of non-violent offending behaviour was not significantly altered when incorporating the risk factors variables (the indirect effect 1st G when applying Khb was non-significant in all models tested). This means that these factors did not have a mitigating effect on the direct association between being a first-generation migrant and the development of non-violent offending behaviour. In other words, the indirect effect of the factors mentioned was not significant in any of the models, so they do not play a role in explaining the higher risk found for 1st generation immigrants compared with the other groups (natives and 2nd generation immigrants).

When violent offences are studied (Table 7), the probability of committing a violent offence is also greater for 1st generation immigrants (the OR is 1.87 in Model 1 vs. 2.13 in Model 4) than for natives. As in the case of non-violent offences, 2nd generation immigrants did not present differences with respect to natives. Other similarities were found with the previous model (non-violent offending): for instance, being *male* had a positive relationship with the development of violent offending, stronger in this case than in the case of non-violent offending, *peer delinquency* was once again the most influential factor in the development of criminal behaviour with violence (although somewhat less so than in the case of non-violent offences—the OR was 8 vs. 11); *neighbourhood disorganisation* exerted a significant influence, as did low *morality* and *self-control*, although in this case there was somewhat more self-control than with non-violent offences; and *father not working* had a significant and direct influence (the OR was 2.6).

Regarding the differences to the previous model, the direct association between the likelihood of committing a violent offence and being a first-generation immigrant, male and with an unemployed father was higher. Moreover, the predictor *school disorganisation* did have a significant relationship with the response variable. In order to examine this, a *t*-test of differences in average scores in *school disorganisation* was carried out comparing the groups that had not committed an offence with those that had (differentiating between with and without violence). The results showed that although the factor score was significantly (sig. 0.000) worst (a higher school disorganisation) in the case related to the group that had committed some type of offence, the difference was greater in the case of violent offences. Therefore, there is a worst score for the school disorganisation factor in the case of the young people who had committed some type of violent offence than in the case of those who had committed non-violent offences. Delving deeper, we found that the greatest difference was in the score related to the first component of this factor (see Table 2, “School disorganisation” factor), which involved *school bonds* (liking and an interest in going to school), and to a lesser extent in those scores that measure delinquency at school (the sale of drugs, fights, and so on). Therefore, it appears that lack of interest and motivation to go to school is a distinctive feature of those who developed violent offending behaviour compared to the other groups analysed. This finding might explain why *school disorganisation* was a significant predictor of violent (Table 7) but not of non-violent (Table 6) offending behaviour. Regarding the influence of the *neighbourhood disorganisation* factor was similar to the development of the two types of delinquency (with and without violence). *Parental control* exerted a significant influence on the output variable, although it turns out that its preventive effect was a little stronger for non-violent offences.

The explanatory power of the violent offences’ model was very similar to that of the non-violence model, only a little higher (pseudo *R*^2^ 0.27). In violent offences, the indirect effect of the *structural explanatory factors*, such as *father not working*, low *parental control*, and high *peer delinquency*, was non-significant at 5% level (khb 1st Generation sig. Indirect effect = 0.08), moreover their influence on the decrease in the OR of the 1st generation variable was minimal (the coefficient changes from 1.87 to 1.83). Something similar happened when the rest of risk factors were included (Model 3 and 4). Therefore, it appears that these risk factors did not significantly mitigate the direct relationship found between being a 1st generation immigrant and the development of violent offending behaviour.

In order to measure the predictive ability of the fitted logistic regression models, with respect to the estimation of the class to which a young person would belong (according to the likelihood of developing offending behaviour without or with violence), a 10-fold cross validation was applied to the original sample. For that purpose, Waikato Environment for Knowledge Analysis (WEKA 3.8) software was used. Considering the confusion matrix obtained for each case (predictive non-violent and violent offending models), 83.2 and 83.6% of the correct classification rate (CCR) was reached on each model, respectively. However, as expected, there was an overfitting of the majority class (non-offending behaviour), with 94% of right classification, and a worse recognition of the minority classes (non-violent and violent offending behaviour) in each model, with 36 and 40% of the right classification rate (“True positive rates”) respectively.

The WEKA-supervised instance filter “Resample” was applied to balance the sample between participants who developed offending (both non-violent and violent) behaviour and those who did not develop any offending behaviour. In this case, undersampling was applied for training, maintaining the instances of the minority class (offending behaviour) and reducing those of the majority class (non-offending behaviour) in order to improve the learning of the minority class The new sample was used to carry out Logistic Regression and a 10-fold validation was also applied.

Although the overall Correct Classification Rate (CCR) decreased somewhat when the class balancer was applied (75.6 and 74.3% vs. 83.2% and 83.6% of the CCR with the original samples, as can be seen in Tables 8, 9 in the “Total cases” row), the minority class (those who developed some offending behaviour) was more clearly recognised (almost 77% of non-violent offending behaviour vs. 74% of non-offending, and 72% of violent offending vs. 76% of non-offending).

**Table 8:** Average proportion of well-classified participants (in total and by class, considering non-offenders vs. non-violent offenders) applying a 10-fold validation.

Group	Sample	
	Original samples	Balanced samples
Non-offending (majority class): True Negative rate	94%	74.6%
Non-violent offending (minority class): True Positive rate	36%	76.7%
Total cases: Correct classification rate: CCR	83.2%	75.6%

**Table 9:** Average proportion of well-classified participants (in total and by class, considering non-offenders vs. violent offenders) applying a 10-fold validation.

Group	Correct classification rate	
	Original samples	Balanced samples
Non-offending (majority class): True Negative rate	94.5%	76.3%
Violent offending (minority class): True Positive rate	40.3%	72.1%
Total cases: Correct classification rate CCR	83.6%	74.3%

Undersampling indirectly achieved a higher representation of the 1st Generation group in the balanced sample, as this group had a higher presence in the minority class (those who reported having engaged in some kind of offending behaviour). The logistic functions obtained when the classes were balanced showed very similar variable coefficients in most variables to those shown in the original samples (Tables 6, 7), except that they resulted in higher odds ratios for the variables reflecting immigrant status (first-generation) and peer influence. Therefore, it could be argued that the results obtained with the LR analysis are quite robust.

## Discussion

The main aim of this study was to explore which of the key elements identified by the literature for explaining differences in juvenile delinquency between immigrant and native youths could be applied in a southern European country such as Portugal. Three important findings have emerged from the research:

1. First-generation immigrants displayed higher crime rates than young Portuguese natives and second-generation immigrants. The difference was significant for both violent and non-violent crimes. These results were maintained with the original samples and also after applying resampling techniques to overcome the existing class imbalance.
2. The risk factors identified by various theories as predictors of the development of offending behaviour and empirically tested in northern European countries are also applicable to southern European countries such as Portugal.
3. There are no significant differences in predictive factors due to the seriousness of the crime (violent vs. non-violent). It was only appreciated that some factors, such as being male or having an unemployed father, exerted a higher influenced on the probability of committed a violent offence. Additionally, school disorganisation, concretely, not having interest or motivation for attending school, is another factor that was directed associated to a higher likelihood of development a violent offence.

The descriptive findings indicate that first-generation immigrant youths have a greater tendency towards both non-violent and violent behaviour when compared with native youths. Specifically, a first-generation immigrant is almost twice as likely to engage in an offending behaviour (with or without violence) than a native, whilst there are no significant differences between natives and second-generation youths. These findings are in line with earlier research according to which there exists a positive correlation between being of immigrant origin and offending behaviour (Killias, 1989; Junger-Tas, 1997, 2001; Marshall, 1997; Kardell and Martens, 2013; Salmi et al., 2015; Bovenkerk and Fokkema, 2016). However, the native and second-generation groups do not present the differences found in other studies (Torgersen, 2001; Killias et al., 2010; Salmi et al., 2015). The crime rate in the immigrants’ country of origin and that of the destination country that receives them could, at least partially, explain some of these differences. Cape Verde and Brazil, the countries from which most immigrants residing in Portugal originate, are amongst those countries with the highest crime rate. In the UNODC violent crime statistics around the world, ranked from the highest to the lowest violent crime rate, Brazil is in 17th position in 2018 (with an index of 27.38 in homicides and 274.32 in serious assaults), Cape Verde is in 56th position (with an index of 6.8 in homicides and 554.47 in serious assaults); whilst Portugal, considered one of the safest countries, is in 166th position (with an index of 0.79 in homicides and 5.86 in serious assaults) (UNODC, 2018).

The results demonstrate that the theoretical construct identified by control and strain theories, as well as by situational action theory, can be applied to adolescents living in Portugal in general, both immigrant and native. It has been found that both structural and individual factors have a direct effect on the committing of juvenile offences (both non-violent and violent). The most influential variable for both types of offending behaviour is *peer delinquency*, followed by *morality* and *self-control*. The marked influence of peer delinquency on juvenile delinquency has been empirically demonstrated by several authors (Wikström and Butterworth, 2006; Wikström and Svensson, 2008; Marshall and Enzmann, 2012; Schils and Pauwels, 2016; Pauwels, 2018). Low *parental social control* directly affects the likelihood of developing offending behaviour (violent and non-violent); although the strength of this influence decreases when structural and individual risk factors are included in the model. It seems, therefore, that whilst *parental control* is indeed important in reducing the propensity for engaging in an offending behaviour, it becomes less so when other factors such as *neighbourhood* and *school environment*, as well as the individual’s *morality* and *self-control,* are considered.

Family socioeconomic factors do not affect the likelihood of developing an offending behaviour (Salmi et al., 2015), except for *father not working*, particularly in the case of violent offences. We have to bear in mind that the data are based on the adolescents’ own assessment and might not constitute accurate information regarding income but do regarding the father’s employment situation, which is a more objective measurement of financial circumstances. With respect to *age* and *gender*, it was found that offending behaviour was more frequent amongst older and male participants, as was expected.

As regards *external factors*, *neighbourhood disorganisation* had a greater significance for non-violent offences, whilst *school disorganisation* was only significant for violent offences. The statistical analysis demonstrates that adolescents who had committed violent offences achieved, on average, a worse score for the latter factor, especially in the component associated with *school bonds*. Therefore, it appears that the young people who had committed violent crimes were often characterised by a greater dislike of and disinterest in going to school. This is in consonance with the findings of other studies that highlight the importance of a lack of educational commitment and a low educational status in explaining juvenile delinquency (Dinovitzer et al., 2009; Aaltonen et al., 2011). With respect to the *neighbourhood disorganisation* factor, the average score was very similar for both types of offence (with and without violence). Then, the high concentration of first-generation immigrant youths in some urban neighbourhoods could explain why this risk factor is significant (Kardell and Martens, 2013; Zimmerman et al., 2015).

The application of khb command showed that the indirect effect of risk factors was non-significant for any type of crime, which means that, as was already concluded by Salmi et al. in a similar study conducted in Finland in 2015, we cannot explain the high prevalence of offending behaviour amongst first-generation immigrant youths by differences in the risk factors analysed, even though these are indeed strong determinants of offending behaviour amongst young people in general.

The predictive ability of the fitted LR models can be considered quite acceptable given the complexity of the problem analysed, especially when the class imbalance problem is solved with some balancing technique.

## Limitations and Suggestions for Future Research

A limitation of this study is that, owing to sample size considerations, there is no evidence regarding the pattern of predictors based on country of origin, with young people from immigrant backgrounds thus being treated as a single group. Even though young immigrants with different origins might be more similar to the majority population than their immigrant parents, more detailed research is still needed that takes these different origins into account.

Another limitation is that the sample sizes of immigrant groups, especially first-generation, were relatively small, which must be considered when drawing conclusions in general terms (although, as explained above, the results were quite similar when cross-validation and class-balancing techniques were applied to the original samples, resulting in a somewhat higher representation of first-generation immigrants).

Nonetheless, the study has contributed to the validity of current approaches by combining the influence of different variables that act simultaneously on delinquent behaviour amongst adolescents.

Hence, as a group, second-generation immigrant youths tend to assimilate native youths’ patterns of juvenile delinquency, as Bersani argued in her study on first- and second-generation immigrant offending trajectories (Bersani, 2014). According to Titzmann et al. (2008), the social processes leading to offending behaviour can be assumed to be the same for both second-generation immigrant and native adolescents. Cultural identity development and social belonging amongst second-generation youths must not be lost sight of in multicultural societies because lack of cultural alignment can increase the propensity for juvenile delinquency in the most vulnerable minors (Aronowitz, 2002; Suárez-Orozco et al., 2009; Neto and Neto, 2014; Van der Gaag, 2019). In this context, the process of social inclusion of immigrant youths should play a crucial role in public policy. The results underline the need to evaluate integration policies in southern Europe more from the perspective of the social and individual development processes of young people of immigrant origin than from an economic perspective. Integration trajectories must be encouraged based on the promotion of structural models that sustain the generation of cultural links with the host society, attachment to the family and participation in social activities.

To summarise, the results of the present study underscore the need to examine in greater depth the explanatory capacity of the risk factors analysed in other southern European countries, as well as the country of origin of young people as an explanatory variable. Likewise, it would be worthwhile to look more closely at those differences between first- and second-generation immigrant youths that contribute to their respective propensities for offending behaviour. For instance, peer delinquency and certain individual factors used as study variables could lead to a reorientation of government intervention when dealing with first-generation migrant youths. All things considered, interventions with immigrant youths should aim to promote their well-being, considering migrant background to be a differential factor in their relationship with crime, and focus on the performance of host countries in order to make an improvement sustainable. Being first-generation immigrant, male and with an unemployed father seems to have a direct association with the likelihood of committing a violent offence. These results suggest the necessity of undertaking a specialised intervention suitable for the aforementioned group. Regarding the second generation youths, addressing peer influence as a central factor in public policy, as well as enhancing cross-cultural moral norms and improving young people’s capacity for self-control should be at the core of public policy. The need to make cities and human settlements in general inclusive and sustainable calls for a reconsideration of integration policies so that they employ evidence-based interventions, for example, by using risk assessment tools based on the different profiles offered by children for more effective risk management.

## Data Availability Statement

The raw data supporting the conclusions of this article will be made available by the authors, without undue reservation.

## Ethics Statement

The studies involving human participants were reviewed and approved by University of Minho’s Ethics Committee. Written informed consent to participate in this study was provided by the participants’ legal guardian/next of kin.

## Author Contributions

All authors contributed to the study conception and design. Material preparation and data collection were performed by SM, PM, and GF-P. The first draft of the manuscript was written by GF-P and analysis was performed by MT-J, and all authors commented on previous versions of the manuscript. All authors contributed to the article and approved the submitted version.

## Funding

This work was supported by the Research Centre in Political Science (UID/CPO/00758/2013), University of Minho, supported by the FCT (Foundation for Science and Technology), and the Portuguese Ministry of Education and Science through national funds, and by the Research Centre on Child Studies (UID/CED/00317/2013), University of Minho, through national funds through the FCT, and co-financed by the European Regional Development Funds (FEDER), and the Competitiveness and Internationalisation Operational Programme (POCI), reference POCI-01-0145-FEDER-007562.

## Conflict of Interest

The authors declare that the research was conducted in the absence of any commercial or financial relationships that could be construed as a potential conflict of interest.

## Publisher’s Note

All claims expressed in this article are solely those of the authors and do not necessarily represent those of their affiliated organizations, or those of the publisher, the editors and the reviewers. Any product that may be evaluated in this article, or claim that may be made by its manufacturer, is not guaranteed or endorsed by the publisher.

## Footnotes

^1^This is shown by the negative sign of the CI limits when the difference is calculated between natives and 1st G immigrants (natives’ proportion – 1st G’ proportion) and the positive sign when the difference is computed between 1st G - 2nd G.
