# Supplementary material for: Analysing the Relationship Between Immigrant Status and the Severity of Offending Behaviour in Terms of Individual and Contextual Factors
Source: Front Psychol. 2022 Jun 15;13:915233. doi: 10.3389/fpsyg.2022.915233 (PMC9240772; doi:10.3389/fpsyg.2022.915233)
Supplement: Supplementary file 1 [file Data_Sheet_1.docx]

# **Appendix A – Original Question Wording**

**Dependent Variable:**

**Offending Lifetime and Past Year Prevalence and Incidence Questions**

**Antisocial behaviour:**

**Graffiti**

Have you ever in your life painted on a wall, train, subway or bus (graffiti)? How often in the last 12 months?

Coded 0 for “no”; 1 for “yes” for the prevalence variables

Measured in number of times for the incidence variables

**Consuming drugs and alcohol**

What kind of things do you usually do in you leisure time? I drink beer/alcohol or take drugs

Coded 0 for “never”; 1 for “sometimes” and 2 for “often”

**Offences without violence:**

**Vandalism**

Have you ever in your life damaged something on purpose, such as a bus shelter, a window, a car or a seat in the bus or train? How often in the last 12 months?

Coded 0 for “no”; 1 for “yes” for the prevalence variables

Measured in number of times for the incidence variables

**Shoplifting**

Have you ever in your life stolen something from a shop or department store? How often in the last 12 months?

Coded 0 for “no”; 1 for “yes” for the prevalence variables

Measured in number of times for the incidence variables

**Theft**

Have you ever in your life stolen something from a person without force or threat? How often in the last 12 months?

Coded 0 for “no”; 1 for “yes” for the prevalence variables

Measured in number of times for the incidence variables

**Dealing with drugs**

Have you ever In your life sold any drugs or help someone selling drugs? How often in the last 12 months?

Coded 0 for “no”; 1 for “yes” for the prevalence variables

Measured in number of times for the incidence variables

**Offences with violence:**

**Extortion**

Have you ever in your life used a weapon, force or threat of force to get money or things from someone? How often in the last 12 months?

Coded 0 for “no”; 1 for “yes” for the prevalence variables

Measured in number of times for the incidence variables

**Group fight**

Have you ever in your life taken part in a group fight in a football stadium, on the street or other public place? How often in the last 12 months?

Coded 0 for “no”; 1 for “yes” for the prevalence variables

Measured in number of times for the incidence variables

**Assault**

Have you ever in your life broken into a building to steal something? How often in the last 12 months?

Coded 0 for “no”; 1 for “yes” for the prevalence variables

Measured in number of times for the incidence variables

**Animal cruelty**

Have you ever in your life hurt an animal on purpose? How often in the last 12 months?

Coded 0 for “no”; 1 for “yes” for the prevalence variables

Measured in number of times for the incidence variables

**Independent Variables:**

**Demographic Questions**

**Gender**

Are you male or female?

Coded 0 for “female”; 1 for “male”

**Age**

How old are you?

Measured in number of years

**Birthplace**

Which country were you born in?

**Mother´s birthplace**

Which country was your (natural) mother born in?

**Father´s birthplace**

Which country was your (natural) father born in?
